# The development and validation of a test of science critical thinking for fifth graders

**DOI:** 10.1186/s40064-015-1535-0

**Published:** 2015-11-26

**Authors:** Ruslan Mapeala, Nyet Moi Siew

**Affiliations:** Institute of Teacher Education, Tawau Campus, KM 32 Jalan Balung, Locked Bag 27, Tawau, 91009 Sabah Malaysia; Faculty of Psychology and Education, Education Block, Universiti Malaysia Sabah, Jalan UMS, Kota Kinabalu, 88400 Malaysia

**Keywords:** Difficulty index, Discrimination index, Science critical thinking test, Validity, Reliability

## Abstract

The paper described the development and validation of the Test of Science Critical Thinking (TSCT) to measure the three critical thinking skill constructs: comparing and contrasting, sequencing, and identifying cause and effect. The initial TSCT consisted of 55 multiple choice test items, each of which required participants to select a correct response and a correct choice of critical thinking used for their response. Data were obtained from a purposive sampling of 30 fifth graders in a pilot study carried out in a primary school in Sabah, Malaysia. Students underwent the sessions of teaching and learning activities for 9 weeks using the Thinking Maps-aided Problem-Based Learning Module before they answered the TSCT test. Analyses were conducted to check on difficulty index (*p*) and discrimination index (*d*), internal consistency reliability, content validity, and face validity. Analysis of the test–retest reliability data was conducted separately for a group of fifth graders with similar ability. Findings of the pilot study showed that out of initial 55 administered items, only 30 items with relatively good difficulty index (*p*) ranged from 0.40 to 0.60 and with good discrimination index (*d*) ranged within 0.20–1.00 were selected. The Kuder–Richardson reliability value was found to be appropriate and relatively high with 0.70, 0.73 and 0.92 for identifying cause and effect, sequencing, and comparing and contrasting respectively. The content validity index obtained from three expert judgments equalled or exceeded 0.95. In addition, test–retest reliability showed good, statistically significant correlations (${\rm r}=0.76, P< 0.01$). From the above results, the selected 30-item TSCT was found to have sufficient reliability and validity and would therefore represent a useful tool for measuring critical thinking ability among fifth graders in primary science.

## Background

Advances in Science and Technology have improved communication, health care, agriculture, educational environment and lifestyle. However, rapid changes in science and technology also come at a cost for humankind and for the environment, and future generations will face even more challenging decisions than the current one. Consequently, teaching critical thinking skills is growing more important as students need to adjust to such change by actively and skillfully conceptualizing, applying, analyzing, synthesizing, and/or evaluating information gathered from, or generated by, observation, experience, reflection, reasoning, or communication (Paul and Elder 2004). Through critical thinking, students can be taught to critically examine different viewpoints on issues concerning the impact of science and technology on everyday life, and evaluate these issues from a societal and environmental perspective. Science educators’ encouragement of students to think critically about science and technology, will also help to develop their analytical skills, as well as their ability to make informed choices in their everyday lives.

In a study using bioethical case study, Gunn et al. (2006) were able to demonstrate how explicit critical thinking instruction, combined with inherently thought-provoking content, could improve critical thinking dispositions, skills, and strategies among Grade Eight students. Another study by Gunn (2007) found that expository comprehension text of science was significantly improved when university students were asked to create critical thinking questions regarding the heart disease text using the six generic question stems. Research evidence has indicated the importance of critical thinking in science lessons and infusing critical thinking should start at the elementary levels. By engaging students in critical thinking from the elementary levels, science educators can lay the foundation for proficient and ethical consumers of scientific change (Gunn et al. 2007).

However, there is little evidence that tests are being used to assess children’ critical thinking in science. Much of the difficulty lies in the lack of tests to assess children’s critical thinking in primary school science classrooms. Ennis (1993) presented a review on critical thinking tests and found that no critical thinking tests were developed with the primary purpose to assess critical thinking in a subject matter area. Most of the developed critical thinking tests such as California Critical Thinking Skills Test (Facione 1992), California Critical Thinking Disposition Inventory (Facione and Facione 1992), Cornell Critical Thinking Test (Ennis et al. 1985) were mostly general-content-based tests. Therefore, a critical thinking test is required to measure how children’s thinking is developed in science lessons. The present study addressed this concern by developing a critical thinking test in science for primary school students.

Attention to critical thinking assessment has recently been emphasized in Malaysian Education Blueprint 2013–2025 (Ministry of Education 2013), resulting in the proliferation of pertinent studies (Daud 2004; Kamrin and Noordin 2008; Rashid and Ismail 2014; Tang et al. 2014). The Malaysian Curriculum and Assessment define the concept of critical thinking as skills needed for a person to systematically assess idea before making a decision (Curriculum Development Centre 2013). However, existing studies (Rashid and Ismail 2014; Tang et al. 2014) are limited where they examine two types of critical thinking skills using instruments that do not adequately assess critical thinking as their primary concern in the context of the primary science classroom. Although critical thinking skill is one of the higher-order thinking skills to be assessed in the revised Malaysian national examinations and school-based assessments, very few standardized instruments with multiple choice test format have been developed to measure critical thinking skill as primary concern in primary science level. Thus, a test needs to be developed that aims to assess critical thinking skills in primary science which is cost effective and easy to be administered.

### The critical thinking test framework

**Fig. 1:**
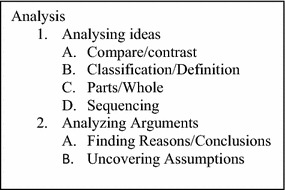
Swartz’s thinking framework of analysis

The critical thinking test is based on Swartz’s (Swartz and Parks 1994; Swartz et al. 2007) thinking framework which combined the Ennis (1993) insights and Bloom’s higher order thinking. This study focusses on Swartz’s *Analysis* framework that incorporates the Ennis-type thinking categories and Bloom’s *Analysis* (Fig. 1). Swartz and Parks (1994) consider analyzing ideas and analyzing arguments as components of analysis. Ennis advocates analyzing ideas such as comparing and contrasting, classifying, sequencing, and predicting as modes of analysis. Whereas *Analysis* from Bloom’s taxonomy is often given as a prompt to solicit a Bloom performance of an act involving differentiating, organizing and attributing (Krathwohl 2002).

According to Piaget’s theory of cognitive development, the developmental stage of the formal operations stage occurs as early as 11 years old until adulthood. Thus, fifth graders at the age 11 are likely to make the transition from concrete operations stage to formal operational thinking. During the transition stage, children develop the ability to think in a logical way (Inhelder and Piaget 1958). According to Wolfinger (2000), the ability to formulate hypotheses was one of the most important processes of logical thought or critical thinking. Formulating hypotheses will gauge student’s thinking on causal order, the sequence in which variables are placed. This sequence determines what is supposedly the ‘cause’ (the independent variable) and what is the ‘effect’ (the dependent variable). In the context of the present study, fifth graders acquired the ability to formulate hypotheses and solve problems through producing several possible methods when they did science. Students were engaged to apply critical thinking skills to analyze information by sequencing, categorizing, identifying cause-and-effect relationships, comparing and contrasting, finding the main idea, and drawing conclusions. Moreover, the Malaysian Ministry of Education has included these skills as components of critical thinking skills to be applied in all Curriculum Standard for Primary Schools (Curriculum Development Division 2012). Thus, assessing critical thinking skills in analysing ideas component of *Analysis* is considered appropriate by considering the cognitive level of fifth graders.

**Fig. 2:**
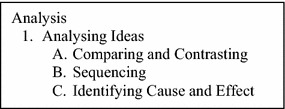
The improved framework for TSCT

In relation to this, the Science critical thinking test developed is based on improved Swartz’s thinking framework that focusses only on comparing and contrasting, sequencing, and identifying cause and effect to the Analysing idea component of *Analysis* (Fig. 2). These three critical thinking skill constructs are also in line with the three types of thinking maps implemented in the program of “I-THINK” introduced by the Ministry of Education: (a) Double Bubble map for comparing and contrasting, (b) Flow Map for sequencing, and (c) Multi Flow map for identifying cause and effect (Curriculum Development Division 2012).

Hence, this study aimed to develop a Test of Science Critical Thinking (TSCT) to measure 11 year-old fifth grader’s thinking skills on comparing and contrasting, sequencing, and identifying cause and effect in science lessons and ascertain the reliability and validity of this test. Comparing and contrasting is the process of looking at similarities and differences in order to reveal important characteristics of two objects, systems, events, processes or concepts. Sequencing is the ability to arrange things in a logical order. Whereas identifying cause and effect is the ability to first look for a cause, or something that makes something else happen, and then look for the effect, or the result of the cause. It is hoped that students are able to tackle new challenges with the ability to think them through and discover solutions once these critical thinking skills are mastered.

## Methods

### Description of TSCT

Ease of administration accompanied by objective scoring procedures was an inducement for researchers to develop a valid and reliable pencil and paper measure of TSCT. A major challenge was to ensure that respondents use critical thinking ability to answer the items on the test. The TSCT thus used a double, multiple choice formats for presenting options for answers, and a choice of critical thinking used for each answer. It was simply to ask “which thinking skills did you use to make this choice?” as an extension of a multiple choice item. One advantage of this multiple-choice-plus-critical thinking choice format was that specific aspects of critical thinking could be covered when scorer was appropriately cautious in the drawing the choice of critical thinking (Ennis 1993). The judgment of critical thinking applied for each answer provided more insights into students’ critical thinking as well as greatly reduced the ‘guess factor’.

Each item required 1.5 min to complete and the whole TSCT would take 45 min. Test requirements in TSCT were the same as for other examinations. A general instructions were printed on the front page of the instrument to not only make the students understand what is required but also encourage them to work hard to complete the TSCT.

### Development of TSCT

The TSCT were constructed based on Swartz’s thinking framework. The TSCT consisted of 55 multiple-choice items with four options measuring critical thinking skill constructs of comparing and contrasting (20 items), sequencing (20 items) and identifying cause and effect (15 items). These items were constructed based on the contents of Year Five Science Curriculum and Assessment Standard Document (Curriculum Development Centre 2013). The contents of the Malaysian syllabus are documented in the Standard Curriculum and Assessment Document. The Year Five Science Curriculum contents are divided into six themes, namely Introduction to the Science, Life Sciences, Physical Sciences, Material Science, Earth and Space Science and Technology and Sustainable Living. Teachers record student achievement for each topic thematically in a document called a Year Five Science Standard Performance Reporting Form (SSPRF) issued by the Ministry of Education. Results from analysis of the SSPRF document in 2014 found that Physical Science as a theme was less mastered by students (Ministry of Education 2014). Therefore, the TSCT items were taken from the Physical Sciences theme, which mostly related to technological products. All these items were reviewed by a group of experts before final versions were designed. The TSCT was presented in the Malay language to ensure that the sample population understood each item before responding. An expert in Malay language proof read the TSCT items. The details of the review of the TSCT items will be further discussed in “Data analysis” section.

In order to elicit the desired critical thinking skills from the students, the stem of each test item was written in such a way that provoked critical thinking. All the stems presented were attached with diagrams, a class of figures or tables to generate alternative choices. Other than selecting the correct response, students also needed to make a choice to determine which critical thinking (comparing and contrasting, sequencing, or identifying cause and effect) used to choose the “right” answer, indicating a specific kind of critical thinking took place. The sample items in each critical thinking skill construct of TSCT are described below to illustrate the item format.

#### Comparing and contrasting

The purpose of this item is to measure student’s thinking about similarities and differences, and drawing rich conclusions from the comparison and contrast made.

##### Sample item

Roziah conduct an experiment to determine the amount of electricity produced by a dry cell battery and an accumulator battery.

Which of the following shows its result after a period of time? 

#### Sequencing

The item measure students’ ability in finding a logical order based on pictures or diagrams given.

##### Sample item

Figure below shows pictures of four battery designs produced to provide energy. 

#### Identifying cause and effect

This item requires students to first look for a cause, or something that makes something else happen. Then students look for the effect, or the result of the cause.

##### Sample item

Figure below shows an electric circuit that is equipped with two bulbs labeled as X and Y. 

### Scoring procedure

Students selected a correct response from the four alternatives and a correct choice of critical thinking used for their response. The students would be awarded one point for the correct response given to both the answer and critical thinking.

### Data analysis

Firstly, in order to identify the construct validity of each item, item analysis was conducted to determine the difficulty index (*p*) and discrimination index (*d*). Accordingly, only item that had a *d* value equaled to or exceeded 0.20 and items of *p* value equaled to or exceeded 0.40 as suggested by Macintosh and Morrison (1969) would be selected for further analysis.

Once the researchers had identified the appropriate items, items were then grouped into three critical thinking skills constructs aimed at determining the internal consistency reliability of each construct in the TSCT. Overall, only the best 10 items were selected for each construct. The Kuder–Richardson 21 (K-R_21_) coefficient of reliability was computed to determine the degree to which dichotomously scored items with about the same difficulty, measure the same construct in order to produce a consistent result (Wood 1960).

Secondly, in order to ascertain the content validity of the selected items in the TSCT, the items were reviewed by subject matter experts. A public university lecturer in the field of thinking skills assessed the three critical thinking skills in TSCT items. A panel of three subject matter experts were involved, one of them was a National Primary School Science Master Trainer, one was Excellent Science Teacher, and one was Primary School Science Master Trainer. Each of the experts was required to confirm several criteria of test content validity namely conformity, accuracy, clarity and appropriateness of the items as issued by the Malaysian Examination Board (MEB) (2013) and provide written comments to improve the items. In this way, subject matter experts reviewed TSCT items for three rounds before they were finalized. Table 1 provides a brief description of each criterion.

**Table 1:** Descriptions of criteria of test content validity

Criterion	Description
Conformity	
Curriculum	The assessed topics were contained in the syllabus
Opportunity	Questions were related to students actual experience
Specifications	Questions met the specifications of the critical thinking skills that were tested
Accuracy and clarity	
Construct	Questions could assess what was required
Context	Students were familiar with the given idea
Component	Language, instruction, diagrams and material stimulus were clear to what pupils needed to do
Suitability	
Difficulty level	The difficulty level of questions were in accordance with tested thinking skills
Important	Item idea and content was important in student’s learning
Fairness	The written items were not bias to the students, gender or specific communities

Source: Malaysian Examination Board (MEB) (2013)

Thirdly, face validity was carried out after the intervention to examine the relevance or transparency of the the TSCT as it appears to the fifth graders (Holden 2010; Gravetter and Forzano 2012). To obtain a measure of face validity of TSCT, a total of 30 students were asked to respond the TSCT items and an unstructured interview with 10 students was carried out on 10 November 2014.

Lastly, test–retest reliability study was conducted separately for a group of fifth graders with similar ability from the same school to determine if the TSCT construct being measured was sufficiently stable and consistent over time with a similar intervention. The same TSCT was given to the same 30 subjects on two separate test administrations, before and after the intervention. The interval between test administrations was 9 weeks. Correlations between these scores were measured using Pearson correlation coefficients.

### Study sample and intervention

The pilot study was conducted with fifth graders in an urban fully government-funded primary school in Tawau, Sabah. The school was selected based on the science subject average grade in the 2014 school assessment test results from Tawau district education office. A class of 30 students in bands 5 and 6 was chosen as study sample. Students in band 5 and 6 were classified as students who could express their creative and innovative ideas, have the ability to make decisions to adapt the demands and challenges in life and talk to acquire and communicate information using their own words (Malaysian Examination Board 2012). Therefore, the selection of these students would allow researchers to get real feedback on TSCT items. Students comprised 18 (60 %) females and 12 (40 %) males aged 11 years old. The number of 30 students was sufficient to determine the reliability of an instrument (Chua 2011).

Prior to administering the test, the practices of thinking-based learning and infusion approaches as advocated by Swartz and Parks (1994) and Swartz et al. (2007) were employed as explicit approach to teaching critical thinking. The science lessons were designed to teach both critical thinking skills and Year Five Physical Science curriculum content simultaneously. The students were introduced explicitly to problem-based learning lessons that were mostly real-world situations familiar to the students. These were developed using the eight steps of Fogarty (1997)’s problem-based learning model: (1) Recognizing the problem, (2) Defining the problem, (3) Triggering ideas through questions, (4) Forwarding the hypothesis, (5) Conducting research, (6) Reviewing the best solution, (7) Choosing the best solution, and (8) Presenting the solution. The students were then prompted to make extensive use of thinking maps on comparing and contrasting, sequencing, and identifying cause and effect to think about the physical science-related problems they were addressing. The types of thinking maps used in the intervention were: (a) Double Bubble Map for fostering the skills of comparing and contrasting, (b) Flow Map for sequencing, and (c) Multi-Flow Map for identifying cause and effect. Students chose and built a thinking map to explain the best solution of the problem presented in the learning lessons. Thus, students would gain benefit from the explicitness of the thinking maps that guide, direct, and incite their critical thinking skills. The intervention consisted of nine lessons of 2 h each, conducted within 9 weeks.

## Findings

The following sections describe preliminary TSCT results in a pilot test on item discrimination and difficulty, internal consistency reliability, content validity, and face validity. The result of test–retest reliability is also included.

### Item discrimination and difficulty

The computed coefficient of item discrimination and difficulty is indicated in Table 2. A total of 30 out of 55 items were selected. All the items in TSCT showed a discrimination index (*d*) range from 0.20 to 1.00, indicating that the items have good discrimination index (Jandaghi 2010). According to Ebel (1979), items with discrimination index greater than 0.20 was an acceptable range in discrimination indexes for item analysis. Similarly, the selected 30 items in TSCT showed a Difficulty Index (*p*) range from 0.40 to 0.60, indicating that the items have relatively good difficulty index (*p*) (Macintosh and Morrison 1969). Then, the 30 items were grouped into three critical thinking skills constructs in order to determine its internal consistency reliability in the subsequent analysis.

**Table 2:** The discrimination index (*d*) and difficulty index (*p*) values of the TSCT

Critical thinking skills construct	Selected item	Discrimination index (*d*)	Difficulty index (*p*)
Comparing and contrasting	CC2	0.63	0.56
	CC7	0.63	0.56
	CC8	0.63	0.56
	CC9	0.25	0.33
	CC10	0.38	0.56
	CC13	0.25	0.33
	CC15	0.63	0.43
	CC17	0.25	0.50
	CC18	0.63	0.56
	CC19	0.63	0.56
Sequencing	S2	0.38	0.56
	S3	0.63	0.56
	S4	0.38	0.56
	S5	0.63	0.43
	S6	0.25	0.50
	S8	0.38	0.56
	S10	0.63	0.56
	S11	0.25	0.38
	S14	0.38	0.56
	S18	0.63	0.56
Identifying cause and effect	CE1	0.60	0.43
	CE2	0.75	0.50
	CE4	0.50	0.50
	CE6	0.25	0.50
	CE7	0.75	0.50
	CE8	1.00	0.50
	CE11	0.87	0.56
	CE12	0.25	0.37
	CE14	0.25	0.50
	CE18	0.75	0.50

*CC* Comparing and contrasting, *S* sequencing, *CE* cause and effect

### Internal consistency reliability

The obtained value of K-R_21_ reliability of the TSCT based upon scores of 30 fifth graders was in the range of 0.70–0.82 (Table 3), indicating that the items have high reliability (Babbie 2001). Consequently, this indicated that all the 30 items contributed to the central test constructs of critical thinking skills on comparing and contrasting, sequencing, and identifying cause and effect.

**Table 3:** The K-R_21_ values of the TSCT

Critical thinking skills construct	Reliability (K-R_21_)
Comparing and contrasting	0.82
Sequencing	0.73
Cause and effect	0.70

### Content validity

Table 4 shows the results of experts’ assessment on the 30 items in TSCT. A high validity index of 0.95 and above was obtained on conformity, accuracy and clarity, and suitability for each item of the TSCT. While the evaluation from the language expert showed that the use of language aspect is appropriate with some improvements.

**Table 4:** The content validity of the TSCT

Expert	Validity percentage (%)		
	Conformity	Accuracy and clarity	Suitability
A	97	95	97
B	98	96	96
C	96	95	97
Validity index	0.97	0.95	0.96

Necessary corrections on the language and appropriateness of the items had been made in accordance with the suggestions provided by the experts.

### Face validity

The expert also agreed with the item complied with the skills tested. Students agreed that the purpose and direction of the questions were clear. Students noted that the use of images or illustration were appropriate and clear. Students also agreed to the use of the font size.

### Test–retest reliability

The obtained Pearson’s product moment correlation coefficient from the test–retest study was ${r}=0.76$ and showed statistically significant correlations (*P* < 0.01). According to Hersen (2004), tests with Pearson correlation coefficients approaching 0.80 demonstrate good test–retest reliability.

## Discussion

This research was a preliminary attempt to develop a TSCT and ascertain its reliability and validity. A 55-item TSCT was developed based on Swartz’s thinking framework for assessing the analysis aspect of critical thinking skill constructs of comparing and contrasting, sequencing, and identifying cause and effect among the fifth graders. The analysis on difficulty index (*p*) and discrimination index (*d*), internal consistency reliability, content validity, face validity and test–retest reliability of the selected 30 items were found to be acceptable and suitable. Difficulty index (*p*) and discrimination index (*d*) obtained by each 30-item of TSCT reached the threshold of an acceptable range. Overall the internal consistency reliability suggested that the TSCT developed was with high reliability in comparing and contrasting, sequencing, and identifying cause and effect.

Each appointed expert greatly approved on the assessment criteria based on conformity, accuracy, clarity and appropriateness of the 30-item TSCT. Some revisions needed to be made in order to improve the language in the test items. Each of the selected 30 items also received positive feedback from a pool of students indicating that the TSCT has good content validity and face validity. The TSCT total scores also demonstrated good test–retest reliability, indicating that the TSCT constructs being measured were relatively consistent over the 9 week intervention.

The principles of using a multiple-choice format and integrating choices of critical thinking skills to each answer offer the potential for developing instruments to measure critical thinking skills that are valid and reliable in science lessons. A 34-item multiple choice forms A and B of California Critical Thinking Skills Test developed by Facione (1992) showed a moderate Kuder–Richardson Reliability of 0.68–0.69. However, the California Critical Thinking Skills Test covered more than one aspect of critical thinking, incorporating interpretation, argument analysis and appraisal, deduction, mind bender puzzles and induction. Study findings showed that a specific-content-based test to check for transfer of critical thinking instruction which is embedded in science subject is possible.

## Conclusion

The findings of this study ascertained that the developed TSCT has relatively high validity and reliability. Overall, TSCT is suitable for testing the three intended critical thinking skill constructs of comparing and contrasting, sequencing, and identifying cause and effect. Thus, the researchers believe that TSCT could be used to assess critical thinking skills of the fifth graders in science.

However, conclusions cannot be drawn from this study as there was no examination on statistically significant differences. For this reason, there is a need for further research. It is recommended that future researchers employ a bigger sample to further validate the instrument. In addition, predictive validity could be evaluated. Researchers could investigate whether fifth grade students’ critical thinking skills can be fostered with a specific intervention programme such as problem-based learning with thinking maps lessons. Other studies may investigate the relationship between Science critical thinking skills and other general critical thinking skills or Science achievement. In addition, specific tests for each or other significant aspects of critical thinking skill could be developed.
